# The Impact of Informal Digital Learning of English (IDLE) on EFL Learners’ Engagement: Mediating Roles of Flow, Online Self-Efficacy, and Behavioral Intention

**DOI:** 10.3390/bs15070851

**Published:** 2025-06-24

**Authors:** Fang Fang, Yaru Meng, Lingjie Tang, Yu Cui

**Affiliations:** 1 School of Foreign Studies, Xi’an Jiaotong University, Xi’an 710049, China; fangfang@sust.edu.cn (F.F.); yucui@stu.xjtu.edu.cn (Y.C.); 2 School of Culture and Education, Shaanxi University of Science and Technology, Xi’an 710021, China

**Keywords:** informal digital learning of English, engagement, flow, online self-efficacy, behavioral intention

## Abstract

In the evolving landscape of online language learning, informal digital learning of English (IDLE) plays a crucial role, particularly among English as a foreign language (EFL) learners. Previous research has investigated the direct impact of IDLE on EFL learners’ engagement. However, little attention has been given to the underlying mechanisms that drive this relationship. To address the gap, this study examined the mediating roles of flow, online self-efficacy, and behavioral intention in the relationship between IDLE and EFL learners’ engagement, with survey data collected from 1194 Chinese EFL learners. Findings reveal that flow, online self-efficacy, and behavioral intention serve as key mediators between IDLE and EFL learners’ engagement in the digital setting. These results offer deeper insights into how informal digital learning influences EFL learners’ engagement in digital contexts, providing valuable implications for both educational theory and digital learning practices.

## 1. Introduction

The advent of digital technologies has catalyzed significant changes in English language education, altering instructional methods and learner experiences, thereby revolutionizing student engagement with English and surpassing the constraints of conventional classroom learning (Lee, 2020; Soyoof et al., 2023). Within this evolving context, informal digital learning of English (IDLE), focusing on English as a foreign language (EFL) learning in digital environments, has emerged as a necessity, as it caters to the diverse learning needs of students, offering flexibility, customization, and real-world language application opportunities that formal education often lacks (G. L. Liu & Wang, 2024). Therefore, researching IDLE is crucial, as it offers insights into enhancing language acquisition by filling the gap between formal instruction and students’ real-world language use (G. L. Liu et al., 2024a).

Furthermore, the significance of affective factors in IDLE has received extensive academic attention (Lee & Drajati, 2019a). Scholars have explored elements like self-efficacy (Su et al., 2018), willingness to communicate (Lee & Drajati, 2019a), well-being (Proietti Ergün & Ersöz Demirdağ, 2023), motivation (Tang et al., 2024), and engagement (Wu, 2023), all of which are crucial for language acquisition within the IDLE context. Engagement is vital for successful online learning because it reflects students’ active striving for learning goals (Peng, 2017), but it is difficult to sustain. Rahman (2021) identified low learners’ engagement as a significant obstacle in online education. Accordingly, there is a pressing need to investigate the factors that affect EFL learners’ engagement, particularly within the context of IDLE (Dixson, 2012; Gamage et al., 2022; Salas-Pilco et al., 2022). Wu (2023) has investigated that IDLE has a positive association with learners’ engagement among EFL college students in online learning. However, the mediating roles of affective factors between IDLE and EFL learners’ engagement remain underexplored, especially in informal language learning settings. To address this gap, our study set out to explore how EFL learners’ engagement is mediated by flow, online self-efficacy, and behavioral intention in IDLE experience. By examining these relationships within the IDLE context, our research aims to contribute to the existing literature and provide valuable insights into the interplay of these factors, ultimately enhancing EFL learners’ engagement in IDLE.

Theoretically, the study draws on multiple frameworks. Flow theory suggests that when learners’ skills match the challenges they face in informal digital language learning, they experience a state of flow, which boosts their engagement, persistence, and enjoyment, leading to better learning outcomes in IDLE (Csikszentmihalyi, 1990). Bandura’s (1977) Social Cognitive Theory asserts that self-efficacy beliefs, including online self-efficacy, play a critical role in shaping engagement and behavior in online educational settings. These beliefs influence both motivation and interaction (Bandura, 2006; C. S. Chang et al., 2014), thereby suggesting a positive association between online self-efficacy and engagement. Ajzen’s (1985) Theory of Planned Behavior argues that behavioral intention, shaped by attitudes, norms, and perceived control, determines behavior. In IDLE, it affects EFL learners’ engagement decisions, commitment, and participation frequency (Ajzen, 1991), with stronger intentions likely leading to increased engagement and better results.

This study contributes in two important ways. First, it advances existing research by exploring the mediating effects of flow, online self-efficacy, and behavioral intention on the relationship between IDLE and EFL learners’ engagement. This investigation offers new insights into the complex processes by which these factors impact EFL learners’ engagement within IDLE contexts. Second, while EFL learners’ engagement in online learning is known to be multifaceted, this study uniquely explores its linguistic dimension, in addition to the more commonly studied cognitive and emotional aspects. This approach provides a more holistic understanding of EFL learners’ engagement within the particular context of IDLE.

## 2. Literature Review

### 2.1. IDLE

Young EFL learners are increasingly engaged in IDLE as opportunities for second language learning increase in out-of-school digital contexts (Lee & Lee, 2021). Based on Benson’s (2011) four dimensions of language learning outside the classroom, IDLE can be characterized as a self-directed activity (locus of control), unstructured activity (formality), and naturalistic activity (pedagogy) that takes place in extramural digital environments (location). EFL learners have been observed participating in both receptive and productive IDLE activities (Lee, 2022; Lai et al., 2015; Lyrigkou, 2019; Sockett, 2014; Sockett & Toffoli, 2012). Receptive activities, such as watching English language YouTube videos and engaging with English content on social media, focus on content comprehension, while productive activities, such as writing comments or interacting with others in English on social media, emphasize content production. By leveraging the flexibility and vast resources of digital platforms, IDLE supports highly personalized and effective language learning (Lee, 2019). This approach not only expands opportunities for language acquisition but also complements traditional language learning methods, making it a versatile and influential tool for fostering language development (Lee, 2020; Li et al., 2021).

Researchers have explored IDLE from multiple angles to understand its complexities and the extensive influence it exerts on language learning. Studies have evaluated the effectiveness of IDLE in improving language skills, emphasizing its substantial effect on learning outcomes (Lee, 2020). Lee (2019) highlighted that understanding the variety and number of IDLE activities is important for how learners perform in English, and these factors might also connect with emotional aspects. Another angle of investigation is that affective variables play a crucial role in IDLE engagement, as demonstrated by Lee and Drajati (2019a). Their findings suggest that fostering positive affective states may enhance the effectiveness of IDLE activities. Lee (2019) found that the diversity of IDLE has a strong relationship with the motivation and self-confidence of Korean EFL learners. Similarly, Lee and Drajati (2019b) reported that there is a relationship between IDLE, receptive informal activities, productive informal activities, grit, motivation, self-confidence, and second language speaking anxiety. Additionally, Lee (2020) revealed that certain profiles, particularly those with maximal engagement, exhibit higher levels of positive affective variables, which may correlate with better language learning outcomes.

Collectively, these studies provide a more refined comprehension of IDLE’s multifaceted role in language education, highlighting its capacity to improve language competency, motivation, engagement, cultural competence, and psychological well-being among learners. However, existing research still has limitations, particularly due to insufficient clarity regarding the mediating roles of affective factors between IDLE and EFL learners’ engagement. In relation to the connection between IDLE and EFL learners’ engagement, limited studies directly address this issue. Wu (2023) examined the interaction between IDLE and student engagement, finding that students’ perceptions of their online learning environment play a crucial role in shaping their engagement levels. In light of this research gap, our study aims to explore the affective mediators that influence the relationship between IDLE and EFL learners’ engagement.

### 2.2. Flow, Online Self-Efficacy, and Behavioral Intention

Flow, as introduced by Csikszentmihalyi in 1990 within the framework of flow theory, is defined as a mental state that fully immerses an individual in an activity. Egbert’s (2003) simplified model of flow and learning highlights that contextual factors, as key antecedents of flow, exert a positive and direct influence on flow. The experience of flow has found significant application in the context of IDLE education, as IDLE learning activities can evoke learners to have meaningful and immersive experiences (Kaye, 2016). This perspective is supported by recent research. Gao et al. (2025) verified the relationship between IDLE activities and flow in the context of EFL learning among Chinese college students. Buil et al.’s (2019) work on clicker usage and Huang et al. (2017), in their study on online games, demonstrated that these elements have a positive impact on flow. In this context, it is reasonable to suggest that IDLE-related activities play a significant role in shaping flow within language learning.

Online self-efficacy refers to an individual’s evaluation of their ability to organize and execute the necessary actions to accomplish specific performance objectives (Bandura, 1977). As noted by Cho et al. (2010), the importance of online self-efficacy becomes even more pronounced in complex and innovative learning environments, such as online education. The positive association between IDLE and online self-efficacy has garnered increasing attention in recent literature. Wu (2023) contends that IDLE might play a crucial role in cultivating online self-efficacy among intermediate Chinese EFL student learners. On a more comprehensive level, Kuan and Lee (2022) hypothesize that the quality of IDLE experiences could have an impact on online self-efficacy. Additionally, Zhang and Li (2021) indicate that IDLE has the potential to elevate learning motivation, which in turn contributes to the enhancement of online self-efficacy. Overall, an extensive review of the relevant literature reveals a robust positive association between IDLE and online self-efficacy.

Behavioral intention pertains to users’ inclination to embrace and utilize technology tools (Cai et al., 2023). In this study, it is defined as learners’ intent to employ digital tools for study within the IDLE framework. Y. J. Chang et al. (2011) investigated the efficacy of augmented reality (AR) in English vocabulary learning, emphasizing the role of technology tools in learners’ behavioral intentions during digital learning. He et al. (2018) showed that digital competence in informal learning environments impacts students’ behavioral intentions. Fan and Wang (2023) examined the behavioral intentions of undergraduates to utilize native Chinese Web 2.0 tools for informal English learning, highlighting the intersection of cultural context and technology in shaping learners’ intentions. Given this context, it is reasonable to assert that, within the IDLE setting, digital tool utilization strongly affects learners’ behavioral intentions.

Based on the above understanding, we propose the following hypotheses to further investigate the impact of informal digital learning of English on flow, online self-efficacy and behavioral intention:

**H1a.**  *Informal digital learning of English has a significantly positive association with flow*.

**H2a.**  *Informal digital learning of English has a significantly positive association with online self-efficacy*.

**H3a.**  *Informal digital learning of English has a significantly positive association with behavioral intention*.

### 2.3. Engagement, Flow, Online Self-Efficacy, and Behavioral Intention

In educational research, engagement is a notion employed to depict students’ degrees of active involvement, curiosity, and purposeful participation in the learning process (Christenson et al., 2012). This concept includes at least three distinct dimensions: behavioral, cognitive, and affective engagement. Within the setting of informal English language activities, the present research utilized the multifaceted concept of EFL learners’ engagement as a structure for examining learners (Wang et al., 2016). Furthermore, while considering involvement in language acquisition (both formal and informal), it is vital to evaluate how much an individual interacts with the language (Arndt, 2023). In addition, the behavioral dimension shows considerable alignment with IDLE behavior, as indicated by factors such as duration, frequency, and the diversity of informal activities. Due to these striking similarities, the inclusion of this dimension in the present research framework is deemed unnecessary. Considering the factors outlined above, the three primary dimensions of EFL learners’ engagement were defined as follows. First, affective engagement encompasses the emotional states that learners experience during informal second language practices. This includes positive emotions like enjoyment and interest, as well as negative ones such as boredom and indifference. Second, cognitive engagement refers to the level of focus that learners direct towards the task at hand. Ultimately, linguistic engagement refers to the extent to which learners intentionally concentrate on analyzing linguistic components and improving their language proficiency (Arndt, 2023). Together, these dimensions offer a thorough understanding of EFL learners’ engagement, emphasizing the active involvement, emotional commitment, and intellectual effort of learners as they engage with and utilize digital platforms for language learning.

The concept of flow, defined as total immersion in an activity, demonstrates its profound influence on EFL learners’ engagement. It posits that individuals experience heightened engagement when they encounter a balance between challenge and skill (Hamari et al., 2016). This balance is crucial in contexts such as game-based learning, where the interplay of flow, immersion, and engagement can enhance learning outcomes (Hamari et al., 2016). The integration of flow theory into models of EFL learners’ engagement reveals that factors such as social support and trust can enhance user experiences in digital platforms (Algharabat & Rana, 2020). This underscores the importance of creating environments that facilitate flow to boost EFL learners’ engagement in online communities. The impact of flow on EFL learners’ engagement is especially significant in gamified educational contexts. Research indicates that flow and emotional engagement are interconnected elements that enhance academic achievement (Özhan & Kocadere, 2020). The ability to achieve flow in informal digital language learning contexts not only enhances EFL learners’ engagement but also promotes deeper learning experiences.

Self-efficacy, defined as an individual’s confidence in their capacity to achieve in specific situations, is a crucial determinant of engagement levels across diverse disciplines. In educational settings, self-efficacy has been linked to learners’ engagement and learning outcomes. Sun and Rueda (2012) conducted a seminal study examining the influence of computer self-efficacy and self-regulation on various dimensions of EFL learners’ engagement—behavioral, emotional, and cognitive—within a distance education context. Their findings suggest that higher levels of self-efficacy are positively correlated with increased EFL learners’ engagement, highlighting the importance of fostering self-efficacy to enhance learning experiences in online contexts. Furthermore, Wu (2023) examined the relationship between EFL learners’ engagement in English language courses and online learning self-efficacy, emphasizing the mediating role of social presence. Higher levels of self-efficacy in online learning have been found to improve EFL learners’ engagement both directly and indirectly through increased social presence, which is essential for creating a positive online learning community. Peng (2017) further advanced this discussion by exploring the impact of EFL learners’ engagement on perceived learning effectiveness within e-learning environments. Their findings suggest that engagement can amplify the positive effects of online self-efficacy on learning outcomes. The relationship between online self-efficacy and EFL learners’ engagement highlights a strong connection, with online self-efficacy emerging as a key factor influencing multiple dimensions of EFL learners’ engagement.

In digital language learning, behavioral intention has a strong positive association with learning effectiveness. In the realm of e-learning, Budu et al. (2018) demonstrated that behavioral intention significantly influences EFL learners’ engagement with e-learning platforms. This finding underscores the necessity of fostering positive behavioral intentions to enhance learners’ engagement in digital learning environments. Furthermore, Alshammari and Babu (2025) proposed that when students perceive a digital tool as useful and simple to use, their satisfaction mediates their intention to engage with it, reinforcing the link between behavioral intention and EFL learners’ engagement. Zhou et al. (2022) investigated teenage EFL learners’ psychological needs and their impact on EFL learners’ engagement and behavioral intention in online course settings. The study reveals that meeting students’ psychological needs is critical for increasing their engagement, which in turn influences their behavioral intentions toward learning. This emphasizes the importance of addressing psychological factors in order to increase engagement and intention in educational settings. Building on the above insights, we propose the following hypotheses to further explore the effects of flow, online self-efficacy, and behavioral intention on EFL learners’ engagement:

**H1b.**  *Flow has a significantly positive association with EFL learners’ engagement*.

**H2b.**  *Online self-efficacy has a significantly positive association with EFL learners’ engagement*.

**H3b.**  *Behavioral intention has a significantly positive association with EFL learners’ engagement*.

### 2.4. The Hypothesized Structural Model

**Figure 1:**
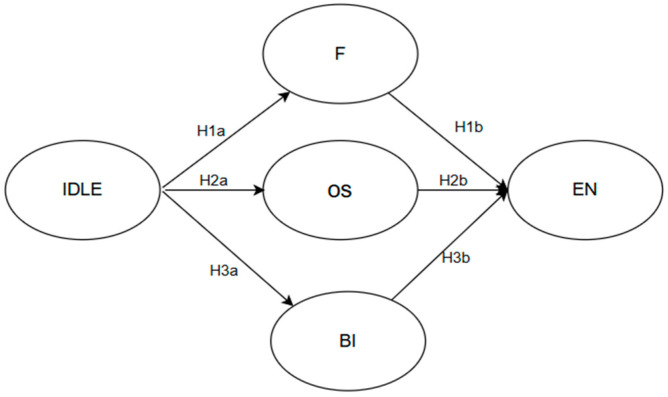
The hypothesized structural model. Note: IDLE = informal digital learning of English; EN = engagement; F = flow; OS = online self-efficacy; BI = behavioral intention.

Figure 1 illustrates the hypothesized structural model, examining the relationships among basic IDLE, mediators (flow, online self-efficacy, and behavioral intention), and engagement. Hypotheses H1a, H2a, and H3a examine the positive associations between IDLE and flow, online self-efficacy, and behavioral intention, demonstrating how IDLE actions support learners’ ability to engage deeply in learning activities. Hypotheses H1b, H2b, and H3b test the influence of flow, online self-efficacy, and behavioral intention on EFL learners’ engagement in IDLE, showcasing their roles in fostering active, cognitive, and emotional involvement in the informal digital learning of English. This model offers an integrated framework for examining the mediating roles of flow, online self-efficacy, and behavioral intention in connecting IDLE activities to EFL learners’ engagement.

### 2.5. Research Questions

Drawing from the previous literature, this study aims to investigate the following questions:

RQ1: Does flow mediate the relationship between IDLE and EFL learners’ engagement, and what specific mediating role does it play?
RQ2: Does online self-efficacy mediate the relationship between IDLE and EFL learners’ engagement, and what specific mediating role does it play?
RQ3: Does behavioral intention mediate the relationship between IDLE and EFL learners’ engagement, and what specific mediating role does it play?

## 3. Methodology

### 3.1. Participants

In the quantitative phase of the study, the participants included 1194 Chinese university EFL students, consisting of 523 males and 671 females aged 18 to 30. Among these 1194 participants, the overwhelming majority (N = 1120, 93.80%) were pursuing undergraduate studies. On closer inspection, this study sample comprised learners majoring in English (N = 41, 3.43%), Humanities (N = 29, 2.43%), social science (N = 195, 16.33%), Science and Engineering (N = 885, 74.12%), and others (N = 44, 3.69%). Also noted is that they all have experience in informal digital language learning. Regarding the specific most-often-used informal digital technologies, the majority preferred music (N = 784, 65.66%) and films (N = 791, 66.25%) in their IDLE activities, while participants (N = 570, 47.74%) used books. Additionally, 445 (37.27%) and 487 (40.79%) participants also chose TV programs (series) and social media (e.g., Wechat, Bilibili), while only 298 (24.96%) participants selected digital games.

### 3.2. Research Instrument

Data were gathered using a revised survey instrument (see Supplementary Materials). The questionnaire was divided into two parts. The first section collected demographic information, including participants’ gender, age, major, and experience with technology use. The second section comprised 26 items adapted from five established and validated scales, assessing IDLE (8 items), engagement (8 items), flow (3 items), online self-efficacy (3 items), and behavioral intention (4 items). Participants responded to all items using a 5-point Likert scale, ranging from 1 (strongly disagree) to 5 (strongly agree).

#### 3.2.1. Informal Digital Learning of English (IDLE)

The IDLE questionnaire, initially developed by Lee and Drajati (2019a), was modified for this study to assess IDLE. It was divided into four sections: form-focused activities, game-based activities, receptive IDLE activities, and productive IDLE activities. The study included eight receptive and productive IDLE items, partially adapted from Lee (2022). An example item was: “I chat with others in English via social media platforms such as Facebook, KaKaoTalk, Line, and WhatsApp.” The Cronbach’s alpha reliability coefficient for the overall scale was calculated at α = 0.824, indicating high reliability.

#### 3.2.2. Engagement

In terms of engagement, we employed a questionnaire adapted from Arndt (2023) to measure EFL learners’ engagement in IDLE. The scale captures various aspects of EFL learners’ engagement with IDLE practices. The engagement questionnaire consisted of 8 items, categorized into three dimensions: affective engagement, cognitive engagement, and linguistic engagement. An example item was: “All in all, I was completely focused on the language during informal digital English learning activities.” The Cronbach’s alpha reliability coefficient for the overall scale in this study was calculated at α = 0.918, demonstrating high reliability.

#### 3.2.3. Flow

With regard to flow, the Hong et al. (2017) questionnaire was validated to evaluate Chinese EFL students’ immersion during informal digital English learning activities. After several rounds of discussion, three items were retained. Sample items include “While using electronic devices (such as WeChat, Weibo, Xiaohongshu, Facebook, Instagram) to learn English, I forgot about everything else.” The Cronbach’s alpha reliability coefficient estimations calculated in the present study revealed high reliability of the total scale (α = 0.864).

#### 3.2.4. Online Self-Efficacy

The questionnaire used in this study, also adapted from Hong et al. (2017), consists of five carefully chosen questions aimed at assessing online self-efficacy, which reflects the belief and confidence in using internet tools for online English learning. After several rounds of discussion, three items were retained. Sample items include “If I came across a new challenge while learning English with electronic devices (such as WeChat, Weibo, Xiaohongshu, Facebook, Instagram), I can always find a strategy to overcome it.” The Cronbach’s alpha reliability coefficient calculated in this study indicated a high level of reliability for the overall scale (α = 0.910).

#### 3.2.5. Behavioral Intention

Behavioral intention involves users’ intention to adopt and use a specific technology tool. To measure learners’ behavioral intention toward social media use in informal digital learning of English, Cai et al.’s (2023) questionnaire was adopted, including four items that were retained. Sample item includes “In the future, I plan to use electronic devices (such as WeChat, Weibo, Xiaohongshu, Facebook, Instagram) to learn English.” The Cronbach’s alpha reliability coefficient estimations calculated in the present study revealed high reliability of the total scale (α = 0.954).

### 3.3. Data Collection and Analysis

Data were collected through an online survey questionnaire distributed to EFL learners in universities. Participants were recruited using convenience sampling methods through various social media platforms and online forums, with informed consent obtained from all participants before survey commencement. The questionnaire included scales to measure the relationship between IDLE and EFL learners’ engagement, as well as constructs related to online self-efficacy, behavioral intention, and flow. Additionally, demographic information such as age, gender, educational background, and frequency of digital English learning usage was collected. In terms of data analysis, it was analyzed in three steps. Firstly, the quantitative analysis included descriptive statistics such as means, standard deviations, and frequencies of survey responses to evaluate central tendencies and variability using SPSS 26.0. The researchers used AMOS 23.0 to test the reliability and validity of the scale and established a structural equation model (SEM) to examine the hypothesized mediation model. SEM enabled the examination of complex relationships between variables and the testing of direct and indirect effects within a single analytical framework.

## 4. Findings

### 4.1. Descriptive Statistics

As indicated in Table 1, the skewness and kurtosis values for all items were below 2 and 10, respectively, suggesting that the dataset follows a normal distribution. The means (M) and standard deviations (SDs) of the observed variables provide insights into the frequency and variability of the latent variables. The SD values for all items ranged from 0.42 to 0.63, reflecting moderate variability in the responses. The M values of IDLE, receptive IDLE activities (M = 3.24, SD = 0.53), and productive ones (M = 2.14, SD = 0.60), slightly below 3.5, indicate that participants held a medium level of positive attitudes towards their informal English learning activities. The M scores for the sub-scales of engagement went beyond 3.50, with affective engagement (M = 3.85, SD = 0.46), cognitive engagement (M = 3.58, SD = 0.42), and linguistic engagement (M = 3.53, SD = 0.43), showing a relatively high level of participants’ engagement in informal second language learning. A close examination of emotions, namely behavioral intention and online self-efficacy, reveal that they were on the slightly high medium level. Meanwhile, the flow emotion is at a slightly high medium level.

### 4.2. Reliability/Validity Checks

The reliability and validity of the scale were assessed to ensure the dataset met the assumptions required for advanced statistical analysis. The Cronbach’s α values for the five scales were 0.82 (IDLE), 0.92 (EN), 0.95 (BI), 0.91 (OS), and 0.86 (F), all exceeding the 0.7 benchmark recommended by Kline (2023), indicating satisfactory internal reliability. For the validity check, we first calculated the standardized factor loadings for each item, along with the composite reliability (CR) and average variance extracted (AVE) values for each variable to evaluate convergent validity. The results indicated that all standardized factor loadings exceeded the 0.5 threshold, with CR values above the 0.5 minimum and AVE values surpassing the 0.5 threshold (Kline, 2023), confirming the establishment of convergent validity (Table 2).

To check for discriminant validity, we followed Henseler et al.’s (2015) recommendation and calculated the Heterotrait-Monotrait Ratio of Correlations (HTMT) for all factors. Since all HTMT values were below 0.90, this indicated that discriminant validity was achieved. To further validate the construct validity, we constructed a measurement model using Amos and assessed its fit by examining six goodness-of-fit indices: the chi-square to degrees-of-freedom ratio (χ^2^/df = 3.787), comparative fit index (CFI = 0.985), incremental fit index (IFI = 0.985), root mean square error of approximation (RMSEA = 0.048), Tucker–Lewis index (TLI), and standardized root mean squared residual (SRMR = 0.029). The data showed a good fit with the measurement model, as all indices met the recommended criteria.

### 4.3. The Structural Model and Hypotheses Testing

Building on the previously established measurement model, we evaluated the full structural model, as indicated in Table 3, which demonstrated a good fit based on the goodness-of-fit indices (χ^2^/df = 3.551, CFI = 0.986, IFI = 0.986, TLI = 0.981, SRMR = 0.028, RMSEA = 0.046). The path analysis results, summarized in Table 4, supported the acceptance of six hypotheses. Specifically, significant positive associations were observed between IDLE and flow (β = 0.79, *p* < 0.001, t = 11.44), OS (β = 0.85, *p* < 0.001, t = 12.31), and behavioral intention (β = 0.79, *p* < 0.001, t = 12.11). Further analysis revealed significant positive associations between participants’ engagement in informal language learning and flow (β = 0.17, *p* < 0.001, t = 4.51), online self-efficacy (β = 0.46, *p* < 0.001, t = 10.99), and behavioral intention (β = 0.23, *p* < 0.001, t = 6.50).

As shown in Table 5, it is important to highlight that the paths “IDLE → F → EN,” “IDLE → OS → EN,” and “IDLE → BI → EN” were both correlated and statistically significant. This indicates that the mediation test’s underlying assumption is fully met. Furthermore, a mediation analysis was conducted to explore the relationship between IDLE and EFL learners’ engagement using a bootstrapped analysis with 5000 samples and 95% confidence intervals in AMOS. The results revealed that the indirect effect of “IDLE → F → EN” was 0.136, with lower and upper bounds of 0.065 and 0.211, respectively. The indirect effect of “IDLE → OS → EN” was 0.388, with lower and upper bounds of 0.300 and 0.484, respectively. The indirect effect of “IDLE → BI → EN” was 0.178, with lower and upper bounds of 0.117 and 0.243, respectively. Since none of the bounds crossed zero, the mediation effects were deemed statistically significant (*p* < 0.01). In other words, IDLE can indirectly affect EFL learners’ engagement through the partial mediators of flow, online self-efficacy, and behavioral intention.

**Figure 2:**
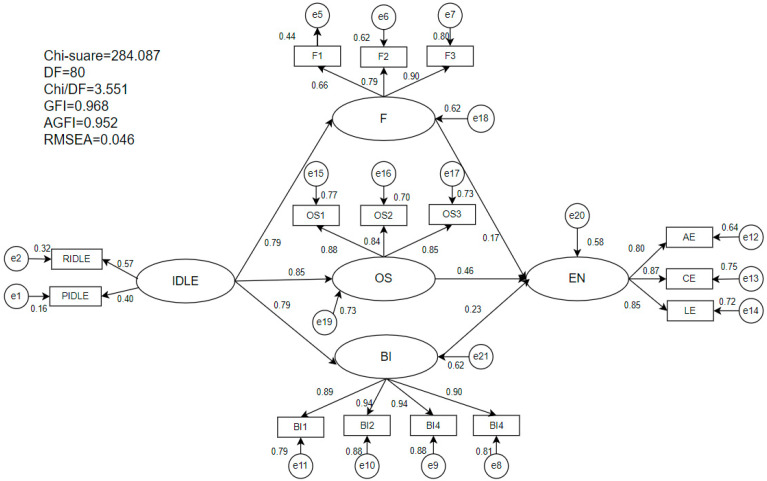
The final structural model. Note: (1) IDLE = informal digital learning of English; EN = engagement; F = flow; OS = online self-efficacy; BI = behavioral intention. (2) R2 (F = 62%; OS = 73%; BI = 62%; EN = 58%).

The R^2^ values shown in Figure 2 indicated that IDLE accounted for 62%, 73%, and 62% of the total variance in flow, online self-efficacy, and behavioral intention, respectively. The combined effects of flow, online self-efficacy, and behavioral intention explained a significant portion (58%) of the variance in EFL learners’ engagement, meaning that the model accounted for 58% of the variation in EFL learners’ engagement. These findings provide support for the explanatory power of the model in understanding how Chinese EFL learners engage in informal digital English learning.

## 5. Discussion

Our research confirms the positive mediating roles of flow, online self-efficacy, and behavioral intention between IDLE and EFL learners’ engagement in digital environments. Building on this core finding, while prior studies have explored foundational mechanisms, our work aligns with and extends Wu’s (2023) demonstration of IDLE’s direct predictive influence on EFL learners’ engagement among EFL learners in digital classrooms. Additionally, this research introduces the dimension of linguistic engagement compared with prior work, aiming to provide a more comprehensive understanding of language learning. The findings hold significant implications for EFL teaching and learning in digital contexts, offering valuable insights into the affective factors that foster active participation and contribute to successful learning outcomes in the IDLE environment.

### 5.1. The Mediating Role of Flow

Regarding the mediating role of flow in response to research question one, our findings show that IDLE predicts flow in the digital environment. This finding is consistent with earlier research, which suggests that flow, defined as complete immersion and participation in an activity, has been associated to improved learning experiences in online environments. For example, it is revealed that factors such as social support and trust in the community of online environments can enhance users’ flow experience in digital platforms (Algharabat & Rana, 2020). Moreover, learners in gamified learning environments much more easily achieve flow experience (Özhan & Kocadere, 2020). Gao et al. (2025) examined the positive mediating roles of flow in the context of IDLE. This finding suggests that achieving flow may mainly depend on the contextual factors of their learning environment. Our study extends this field by empirically validating IDLE as predictors of flow among EFL learners in the context of digital learning. Moreover, flow has been identified as a key predictor of cognitive, emotional, and linguistic engagement in online learning contexts. This finding aligns with previous research by Gao et al. (2025), Hamari et al. (2016), Santosa (2015), and Özhan and Kocadere (2020), which highlights the significant role of flow in influencing various aspects of EFL learners’ engagement, particularly cognitive and emotional dimensions. Our study also focuses on the linguistic dimension of engagement, offering a deeper understanding of how EFL learners’ engagement is linked to language acquisition. This connection is consistent with the findings of Li et al. (2021) and H. Liu and Song (2021). Li et al. (2021) explored the impact of flow on language learning motivation and EFL learners’ engagement in digital learning settings, demonstrating how a state of flow improves learners’ attention to language content. H. Liu and Song (2021) show that when learners attain a flow state, they become more engaged in language-related tasks. These studies, taken together, show that learners who are in flow exhibit higher levels of language engagement in IDLE.

Flow mediates as an attention intensifier, efficiently filtering out distractions that often derail IDLE. It allows students to thoroughly immerse themselves in language-rich material, such as English films or online games, which greatly improves their attention. This increased attention is critical for effective learning and long-term engagement. Flow also serves as a powerful motivation enhancer. During a flow state, learners experience enjoyment and accomplishment, which ignites an internal drive. This sense of accomplishment motivates them to explore a diverse range of digital English learning resources, propelling them to engage with IDLE on a continuous basis. Finally, flow functions as an enabler for skill development. It allows learners to quickly absorb new language knowledge and apply it in real time. This efficient learning process builds their confidence in using English, encouraging them to engage more deeply with IDLE activities and further their language proficiency. Collectively, these roles emphasize the critical function of flow as a mediator, strengthening the connection between IDLE and EFL learners’ engagement while also underscoring its vital contribution to the effectiveness and flexibility of the language learning process.

The findings emphasize several important pedagogical implications for promoting flow in informal digital learning settings. First, integrating storytelling, especially digital storytelling, as a pedagogical tool engages learners in a student-centered way, enhancing motivation and enabling personalization and communication. Second, social interaction is crucial, as promoting social connections among learners boosts engagement and immersion in such environments, with collaboration and dialogue facilitating deeper learning for achieving flow. Third, educational practices need to adapt to the digital landscape, with a call for redesigning educational frameworks to incorporate digital-enabled collaboration, and educators are urged to experiment with new digital-based formats and strategies. Additionally, teacher training in using digital storytelling and innovative techniques is important, like the T-Story project’s goal of enhancing educators’ storytelling skills to better engage students in informal settings. Overall, a multifaceted approach involving these elements is necessary to create engaging and immersive learning experiences for achieving flow.

### 5.2. The Mediating Role of Online Self-Efficacy

Regarding the mediating role of online self-efficacy in research question two, our discussions are as follows. IDLE significantly predicts online self-efficacy in digital learning. This finding is in line with existing research indicating that the higher the quantity and quality of IDLE experiences, the greater the improvement in their self-efficacy in online learning contexts. This aligns with the notion that effective IDLE can enhance learners’ confidence and learning motivation in their abilities to succeed in online settings, which subsequently boosts online self-efficacy, further supporting the idea that informal digital learning experiences are crucial for developing self-efficacy in online learning contexts (Cui & Meng, 2023; Kuan & Lee, 2022; Wu, 2023; Zhang & Li, 2021). Our research advances this field by providing empirical evidence that IDLE serves as a predictor of online self-efficacy among EFL learners in informal digital environments. Additionally, our results demonstrated a direct link between online self-efficacy and EFL learners’ engagement within the context of IDLE, aligning with previous studies that emphasize the positive influence of self-efficacy on EFL learners’ engagement (Derakhshan et al., 2023; G. L. Liu et al., 2024b; Wu, 2023). This implies that higher online self-efficacy levels can improve EFL learners’ engagement. These results are in line with Bandura’s self-efficacy theory, which holds that people who have a high level of confidence in their skills are more likely to take on difficult tasks. For the engagement in this study, Sun and Rueda (2012) illustrate online self-efficacy’s pivotal role in shaping cognitive and emotional facets of engagement. In terms of linguistic engagement, Wu (2023) discovered that students who score higher on online self-efficacy promote greater participation in language-related tasks during IDLE. Su et al. (2018) demonstrated that more active participation in language-related activities was correlated with higher levels of self-efficacy. These studies show that increased EFL learners’ engagement in linguistic activities in online English learning environments is linked to higher levels of online self-efficacy.

Online self-efficacy serves as a mediator and enhancer of metacognitive skill in IDLE, allowing learners to use metacognitive methods effectively in digital English learning. Learners with high self-efficacy are more likely to organize their studies, keep thorough records of their progress, and reflect on their mistakes. They actively dissect language structures, contrast usage examples, and engage deeply with texts. Such immersive interaction enhances language comprehension, fortifies knowledge retention, and aids in applying skills to novel contexts. As a proficiency catalyst, online self-efficacy allows learners to approach digital English learning materials with confidence. This confidence encourages them to actively practice grammar, expand their vocabulary, and engage consistently with various linguistic tasks. Through such regular interaction with different language activities, learners can promote their overall language development. Online self-efficacy also acts as an emotional stabilizer. It cultivates positive attitudes towards digital English learning, effectively reducing feelings of anxiety and self-doubt. By equipping learners with the emotional resilience necessary to navigate challenges, it maintains their passion and guarantees ongoing, active engagement in digital English learning activities. Collectively, these roles highlight the critical role of online self-efficacy as a mediator, not only reinforcing the connection between IDLE and EFL learners’ engagement but also underscoring its profound impact on enhancing EFL learning. High levels of online self-efficacy contribute significantly to the resilience, adaptability, and success of learners as they navigate digital environments to develop their English proficiency.

The results reveal three major pedagogical implications for strengthening online self-efficacy within informal digital learning settings. First, fostering social interaction and encouraging self-directed learning are crucial strategies for enhancing online self-efficacy. To support this, educators and platform designers can incorporate features such as discussion boards, collaborative projects, and a variety of learning materials. These foster a sense of connection with peers and autonomy, which are vital for learners to believe in their online learning success. Second, online self-efficacy could be considered a crucial catalyst for enhancing EFL learners’ engagement. Students might be assisted in choosing activities that effectively combine challenge and personal interest. When tasks are suitably hard and align with personal preferences, learners are more inclined to feel a sense of achievement, hence enhancing their online self-efficacy. Third, cultivating online self-efficacy in informal digital contexts necessitates the adoption of measures that mitigate external demands while providing supportive resources. Facilitating adaptable educational trajectories and effective progress-assessment instruments can assist in attaining this objective. These measurements maintain the autonomous and investigative nature of informal learning, enabling learners to cultivate a robust and lasting feeling of online self-efficacy.

### 5.3. The Mediating Role of Behavioral Intention

In relation to the mediating role of behavioral intention addressed in research question three, our discussion is outlined as follows. IDLE significantly predicts behavioral intention in digital settings. This finding aligns with multiple research studies. Fan and Wang (2023) found that learners’ positive perception of digital tools was positively related to their behavioral intention to use these tools, indicating that IDLE experiences can shape learners’ behavioral intentions. He et al. (2018) incorporate digital competence as a critical factor influencing students’ behavioral intentions toward digital informal learning. In addition, Lee and Sylvén (2021) demonstrated that the frequency of IDLE predicted can influence learners’ behavioral intention to communicate in English. Our study contributes to this field by providing empirical evidence that IDLE experiences have a positive association with behavioral intention among EFL learners in informal digital environments. Moreover, behavioral intention has been acknowledged as an important predictor of EFL learners’ engagement within digital learning contexts. This relationship is consistent with prior research. For example, Budu et al. (2018) highlighted that behavioral intention significantly impacts EFL learners’ engagement with e-learning platforms. Alshammari and Babu (2025) suggest that when students perceive a tool as useful and easy to use, their satisfaction mediates their intention to engage with it, thereby reinforcing the connection between behavioral intention and EFL learners’ engagement. Furthermore, our study contributes to this field by empirically demonstrating that behavioral intention positively predicts linguistic engagement, which is consistent with previous research, in addition to the common cognitive and emotional engagement. Fan and Wang (2023) found that undergraduates with a positive perception of Web 2.0 tools’ benefits for English skills have a stronger intention to use them, increasing digital language-related activity participation. Moradi et al. (2023) found that when Chinese undergraduates used language strategies more often, it led to them taking part more actively in English conversations. Learners who are satisfied with intelligent chatbots are more likely to continue using these conversational tools to improve their learning activities. These studies imply that a stronger behavioral intention in language-related activities is associated with greater linguistic engagement. This link demonstrates the importance of behavioral intention in increasing the depth and quality of engagement in language learning.

In IDLE, behavioral intention serves as a mediating enabler of strategic thinking. It gives students the ability to make flexible study schedules, evaluate their progress informally, and own up to mistakes. It improves language mastery, retention, and application by pushing students to examine linguistic structures, contrast usage, and gain a deep understanding of texts. Furthermore, behavioral intention serves as an action driver in IDLE, motivating students to study digital content, practice grammar, increase their vocabulary, and participate in language tasks. It is driven by the emotion of self-fulfillment. It encourages regular engagement with casual activities, gradually improving their ability to communicate digitally. In addition, behavioral intention is an important psychological supporter. It promotes positive mindsets, allowing students to see challenges as opportunities for growth and reducing anxiety associated with unstructured learning. By providing them with the mental strength to deal with issues such as inconsistent digital content, it maintains their enthusiasm and ensures ongoing engagement in digital English learning. Collectively, these roles highlight the critical role of behavioral intention as a mediator, not only reinforcing the connection between IDLE and EFL learners’ engagement but also underscoring its profound impact on enhancing language acquisition. High magnitudes of behavioral intention significantly foster learners’ self-directed learning, endow them with greater motivation, and facilitate their success as they maneuver through digital platforms to elevate their English language skills.

The research outcomes underscore three essential pedagogical considerations for improving behavioral intention in the realm of informal digital learning. Firstly, social-cognitive engagement: incorporate real-time chat during online sessions and peer-to-peer mentoring programs. When learners feel connected and supported through social interactions, they develop a positive attitude toward the platform, increasing their intention to use it for future learning. Secondly, goal-oriented task design: help learners set clear goals and break them into manageable tasks. As learners achieve these tasks and see progress, their online self-efficacy improves, strengthening their intention to continue engaging with the platform to reach long-term goals. Thirdly, user-experience optimization: ensure straightforward navigation, a minimalist design, and instant feedback on actions. A positive, glitch-free user experience creates a favorable perception of the platform, driving learners’ intention to reuse it for a smooth learning experience.

## 6. Conclusions

This study set out to explore the mediating functions of flow, online self-efficacy, and behavioral intention in the relationship that exists between IDLE and the engagement of EFL university students within a digital context. The results of our research confirm that flow, online self-efficacy, and behavioral intention fully mediate the association between IDLE and EFL learners’ engagement. Flow enables learners to become deeply engrossed in IDLE-related activities. This state of absorption triggers the motivation to explore and make use of a wide variety of digital English learning resources, which in turn fosters EFL learners’ engagement. Students develop a greater sense of confidence in their ability to succeed in the online learning community when they fully engage in IDLE activities. A higher level of online self-efficacy has a positive impact on students’ online learning experiences, as it allows them to approach various tasks and uncertainties in digital learning environments with self-assurance, thereby facilitating more effective learning and enhancing engagement. Behavioral intention serves as a vital mediator in the relationship between IDLE and EFL learners’ engagement. Behavioral intention, which is motivated by the desire for self-fulfillment, is an essential component that fully sustains engagement. It gives students the tools they need to deal with the difficulties and intricate relationships found in IDLE settings. Collectively, the mediating functions of flow, online self-efficacy, and behavioral intention clarify how they influence the IDLE experience and, consequently, affect the engagement levels of Chinese university EFL students. These results present beneficial insights for educators and policymakers to devise impactful strategies to heighten learner participation in informal digital contexts.

There are limitations to the current investigation. First of all, relying solely on online surveys to collect quantitative data would have resulted in incomplete data because it might not have fully captured the participants’ varied perspectives and experiences. Second, the study leaves out important techniques that could offer deeper and more complex insights into students’ experiences with flow, online self-efficacy, and behavioral intention, such as interviews, observations, or learner diaries. Future studies should investigate how to overcome these constraints by integrating various data collection methods. Additionally, the dynamics of flow, online self-efficacy, and behavioral intention in IDLE. longitudinal studies could provide insights into the long-term trajectories of these constructs, uncovering key developmental phases or external influences that affect learner engagement over time. These developments would help us gain a more profound insight into the development of flow, online self-regulated learning efficacy, and behavioral intention within informal digital learning environments. By doing so, they would set the stage for the creation of more focused and successful educational strategies.

## Figures and Tables

**Table 1:** Descriptive statistics and factor loading (In CFA).

Factors		M	SD	Kurtosis	Skewness	Factor Loading	α (>0.7)
IDLE	RIDLE	3.239	0.530	0.472	−0.207	0.914	0.824
	PIDLE	2.139	0.603	0.173	0.637	0.623	
EN	AE	3.851	0.462	0.711	−0.25	0.804	0.918
	CE	3.579	0.424	0.47	0.019	0.866	
	LE	3.526	0.427	0.697	−0.093	0.848	
BI	BI1	3.660	0.600	0.647	−0.397	0.887	0.864
	BI2	3.700	0.570	0.701	−0.452	0.936	
	BI3	3.690	0.563	0.674	−0.397	0.941	
	BI4	3.690	0.586	0.685	−0.402	0.902	
F	F1	3.040	0.634	0.493	0.067	0.662	0.954
	F2	3.150	0.600	0.421	0.085	0.783	
	F3	3.330	0.538	0.405	0.157	0.901	
OS	OS1	3.500	0.522	0.102	0.049	0.832	0.910
	OS2	3.520	0.535	0.211	−0.055	0.896	
	OS3	3.540	0.517	0.19	−0.021	0.913	

Note: IDLE = informal digital learning of English; RIDLE = receptive informal digital learning of English; PIDLE = productive informal digital learning of English; EN = engagement; AE = affective engagement; CE = cognitive engagement; LE = linguistic engagement; F = flow; OS = online self-efficacy; BI = behavioral intention.

**Table 2:** Convergent validity and discriminant validity.

		AVE (>0.5)	CR (>0.7)	HTMT (<0.9)				
				IDLE	EN	BI	F	OS
1	IDLE	0.612	0.753	0.782				
2	EN	0.705	0.878	0.537	0.840			
3	BI	0.841	0.955	0.498	0.635	0.917		
4	F	0.621	0.829	0.481	0.612	0.616	0.788	
5	OS	0.776	0.912	0.479	0.691	0.640	0.649	0.881

Note: IDLE = informal digital learning of English; EN = engagement; F = flow; OS = online self-efficacy; BI = behavioral intention.

**Table 3:** Model fit Indices.

	X^2^/df	CFI	IFI	TLI	RSMEA	SRMR
The measurement model	3.787	0.985	0.985	0.979	0.048	0.0289
The structural model	3.551	0.986	0.986	0.981	0.046	0.0283
Cutoff values (Kline, 2023)	<5	>0.90	>0.90	>0.90	<0.10	<0.08

Note: CFI = comparative fit index; IFI = incremental fit index; TLI = Tucker–Lewis index; RMSEA = root mean square error of approximation; SRMR = standardized root mean squared residual; PNFI = parsimony normed fit index.

**Table 4:** Hypotheses test results.

Path	*β*	*p*	t-Value	Results
IDLE → F	0.790	***	11.44	accepted
IDLE → OS	0.852	***	12.31	accepted
IDLE → BI	0.786	***	12.11	accepted
OS → EN	0.455	***	10.99	accepted
BI → EN	0.227	***	6.50	accepted
F → EN	0.172	***	4.51	accepted

Note: (1) *** *p* < 0.001; (2) IDLE = informal digital learning of English; EN = engagement; F = flow; OS = online self-efficacy; BI = behavioral intention.

**Table 5:** Goodness-of-fit indices of the measurement models.

Mediation Paths	95% Confidence Interval		*p* (Two-Tailed Significance)	Indirect Effect	Results
	Lower Bound	Upper Bound			
IDLE → F → EN	0.065	0.211	0.000	0.136	accepted
IDLE → OS → EN	0.300	0.484	0.000	0.388	accepted
IDLE → BI → EN	0.117	0.243	0.000	0.178	accepted

Note: IDLE = informal digital learning of English; EN = engagement; F = flow; OS = online self-efficacy; BI = behavioral intention.

## Data Availability

The data presented in this study can be made available upon reasonable request from the corresponding author.

## Supplementary Materials

The following supporting information can be downloaded at https://www.mdpi.com/article/10.3390/bs15070851/s1.
