# Supplementary material for: The Impact of Informal Digital Learning of English (IDLE) on EFL Learners’ Engagement: Mediating Roles of Flow, Online Self-Efficacy, and Behavioral Intention
Source: Behav Sci (Basel). 2025 Jun 24;15(7):851. doi: 10.3390/bs15070851 (PMC12292326; doi:10.3390/bs15070851)
Supplement: Supplementary file 1 [file behavsci-15-00851-s001.zip › behavsci-3644029-supplementary.pdf]

Supplementary

English version	Chinese version
**IDLE**	
Receptive IDLE	
I read English entertaining contents (e.g. shopping ads, comics, travel publications, movie reviews) online.	我在网上阅读英语娱乐内容（例如购物广告、漫画、旅游出版物、电影评论）。
I watch YouTube clips.	我观看 YouTube 短片
I listen to English language news programs online or on TV.	我在网上或电视上收听英语新闻节目。
I watch English comics online or on TV.	我在网上或电视上观看英语电影。
Productive IDLE	
I chat with others in English via social media such as Facebook, KaKaoTalk, Line, and WhatsApp.	我通过电子设备（如微信、微博、小红书、Facebook、KaKaoTalk、Line 和 WhatsApp 等）用英语与他人聊天。
I talk with native English speakers (e.g. American, British, Australian) via social media.	我通过电子设备（如微信、微博、小红书、Facebook、KaKaoTalk、Line 和 WhatsApp 等）与英语母语者交谈。
I share English contents online.	我在网上分享英文内容。
I search for information in English.	我用英语搜索信息。
**Engagement**	
Affective Engagement	
All in all, informal digital English learning activities were entertaining	总的来说，非正式英语数字学习平台活动是有趣的。
Informal digital English learning activities were very interesting to me.	非正式英语数字学习平台活动内容对我来说是有趣的。
I really liked informal digital English learning activities.	我喜欢非正式英语数字学习平台活动。
Cognitive Engagement	
During informal digital English learning activities, I concentrated hard on it.	在非正式英语数字学习平台活动中，我是专心的。
During informal digital English learning activities, I was completely focused on the content.	在非正式英语数字学习平台活动的内容中，我当时完全专注于内容本身。
Linguistic Engagement	
During informal digital English learning activities, I was completely focused on the trying to understand every single word	在非正式英语数字学习平台活动的内容中，我当时完全专注于理解每一个单词。
All in all, I was completely focused on the language during informal digital English learning activities.	在非正式英语数字学习平台活动的内容中，我当时完全专注于这门语言。
All in all, I thought a lot about the language during informal digital English learning	在非正式英语数字学习平台活动的内容中，我对语言思考了很多。

activities.	
**Behavioral Intention**	
I intend to use electronic devices (such as WeChat, Weibo, Xiaohongshu, Facebook, Instagram) to enhance my language learning in the future.	未来我打算使用电子设备(如微信、微博、小红书、Facebook、Instagram)学习英语。
I intend to use the learning contents in electronic devices (such as WeChat, Weibo, Xiaohongshu, Facebook, Instagram) to enhance my learning.	我打算使用电子设备(如微信、微博、小红书、Facebook、Instagram)的学习内容促进我的英语学习。
I intend to use electronic devices (such as WeChat, Weibo, Xiaohongshu, Facebook, Instagram) to enhance my learning intention.	我打算使用电子设备(如微信、微博、小红书、Facebook、Instagram)提高我的学习意愿
I intend to use electronic devices (such as WeChat, Weibo, Xiaohongshu, Facebook, Instagram) as an autonomous learning tool.	我打算将电子设备(如微信、微博、小红书、Facebook、Instagram)作为一个自主学习的工具。
**Flow**	
While using electronic devices (such as WeChat, Weibo, Xiaohongshu, Facebook, Instagram) to learn English, I forgot about everything else.	在使用电子设备(如微信、微博、小红书、Facebook、Instagram)学习英文的时候,我忘记了其他所有事情。
I was immersed in English learning while using electronic devices (such as WeChat, Weibo, Xiaohongshu, Facebook, Instagram).	使用电子设备(如微信、微博、小红书、Facebook、Instagram)学习英语时,我完全沉浸其中。
I felt very concentrated on my English learning while using electronic devices (such as WeChat, Weibo, Xiaohongshu, Facebook, Instagram).	在使用电子设备(如微信、微博、小红书、Facebook、Instagram)学习英语时,我感到非常专注。
**Online Self-efficacy**	
If I study English hard, I can solve any problem while using electronic devices (such as WeChat, Weibo, Xiaohongshu, Facebook, Instagram) to learn.	如果我努力学习英语,我会通过电子设备(如微信、微博、小红书、Facebook、Instagram)解决任何问题。
If there is new English content on electronic devices (such as WeChat, Weibo, Xiaohongshu, Facebook, Instagram), I have the confidence to master it.	如果电子设备(如微信、微博、小红书、Facebook、Instagram)有新的英语内容,我有信心能够掌握它。
If I came across a new challenge while learning English with electronic devices (such as WeChat, Weibo, Xiaohongshu, Facebook, Instagram), I can always find a strategy to overcome it.	如果我在使用电子设备(如微信、微博、小红书、Facebook、Instagram)学英语时遇到了新的挑战,我总能找到方法来克服它。
